# Imperial Granum

**Published:** 1884-01-20

**Authors:** 


*Imperial Granum.*—Read carefully the advertisement of this Food preparation. We regard it as a most excellent preparation.

*Treatment of Wounds*—As based on Evolutionary Laws, by C. Pitfield Mitchell, member of the Royal College of Surgeons, etc. New York: J. H. Vail & Co, 1883. Price, 50 cents, 29 octavo pages, cloth.

*Harter's Iron Tonic.*—See the advertisement of this preparation, commencing in this number of our Journal.

SEE the advertisement of the Bromo-Chemical Co , commencing with this number of our Journal.

*Tongaline.*—Our readers should notice carefully the formula of Tongaline as prepared by A. A. Mellier, of St. Louis. The combination seems to be admirably adapted to neuralgic and rheumatic affections.

